# Effect of mesenchymal stem cells and polyvinyl alcohol-coated selenium nanoparticles on rats with Alzheimer-like phenotypes 

**DOI:** 10.22038/ijbms.2024.76242.16495

**Published:** 2024

**Authors:** Siamak Shahidi, Sara Soleimani Asl, Bahareh Gholamigeravand, Simin Afshar, Nasrin Hashemi-Firouzi, Alireza Samzadeh-Kermani, Mahsa Majidi, Kimia Amiri

**Affiliations:** 1 Neurophysiology Research Center, Hamadan University of Medical Sciences, Hamadan, Iran; 2 Department of Anatomy, School of Medicine, Hamadan University of Medical Sciences, Hamadan, Iran; 3 Department of Chemistry, Faculty of Science, University of Zabol, Zabol, Iran; 4 School of Medicine, Hamadan University of Medical Sciences, Hamadan, Iran

**Keywords:** Alzheimer disease, Memory, Polyvinyl alcohol, Selenium, Stem cells, Streptozocin

## Abstract

**Objective(s):**

Mesenchymal stem cell (MSC) transplantation represents a promising approach for treating Alzheimer’s disease (AD). These stem cells, however, have a short lifespan following transplantation into recipient animals. Selenium nanoparticles, due to their size, aid in drug delivery for brain disorders. This study investigated the therapeutic effect of MSCs and polyvinyl alcohol (PVA)-coated selenium nanoparticles (SeNPs) in a rat model of AD.

**Materials and Methods:**

An Alzheimer-like phenotype was induced through intracerebroventricular (ICV) administration of streptozotocin (STZ). Rats were assigned to five groups: control, Alz (STZ; 3 mg/kg, 10 μl, ICV), Alz+stem cell (ICV transplantation), Alz+SeNP (0.4 mg/kg, orally), and Alz+stem cell+SeNPs. The ICV administration of STZ mimicked some aspects of AD in the Alz groups. SeNPs were administrated for 30 days following STZ administration. The novel object recognition (NOR) and passive avoidance learning (PAL) tests were used to evaluate cognition and memory. Oxidative stress biomarkers and brain-derived neurotrophic factor (BDNF) were assessed by biochemical analysis, ELISA kits, and Congo red staining, respectively.

**Results:**

The combined therapy of PVA-coated SeNPs and MSC transplantation was more effective in enhancing memory reacquisition compared to either SeNPs or MSCs alone. The use of stem cells in conjunction with PVA-coated SeNPs significantly boosted anti-oxidant capacity.

**Conclusion:**

The results suggest that the joint treatment with PVA-coated SeNPs and MSCs offers considerable neuroprotection against AD in animal models.

## Introduction

Mesenchymal stromal/stem cells (MSCs) originate from the multipotent progenitor cells found in various human tissues and organs, which are utilized in cell therapy (1). Among these sources, adipose tissue is frequently used in clinical studies; however, its applications have yielded varying and sometimes conflicting results (2). 

The blood-brain barrier’s selective system plays a crucial role in regulating the central nervous system (CNS) homeostasis and protecting the CNS against toxins, pathogens, inflammation, injury, and diseases (3). However, the MSCs can migrate across endothelial cells through either the paracellular or transcellular pathways, subsequently homing back preferentially to sites of inflammation or injury in the brain to exert their therapeutic effects (4). This capability has led to the exploration of stem cells for the treatment of brain disorders, such as traumatic brain injury, stroke, Parkinson’s disease, and Alzheimer’s disease (AD) (1, 4-6).

However, treating complicated diseases, such as AD needs targeting multiple factors involved in the disease process through combinatorial therapies (5). Recent developments in combined therapy for AD have shown promising results, offering hope for better future prevention or treatment strategies (7). Neuronal death, particularly in the CNS areas, such as the hippocampus, is a hallmark of AD (8). Oxidative stress and neuroinflammation are associated with the pathology of AD progression, leading to memory impairment (9). The potential of stem cell therapy in treating AD has been acknowledged (10). However, unfavorable conditions such as oxidative stress and inflammatory agents in the tissue can eliminate most transplanted stem cells in the early days following transplantation, thereby reducing their effectiveness. Enhancing tissue conditions with anti-oxidant or anti-inflammatory agents may improve stem cell survival and the efficacy of cell therapy (11). 

Various animal models are utilized to study AD. The intracerebroventricular (ICV) injection of streptozocin (STZ) is a commonly used method to simulate sporadic AD (12). The ICV injection of STZ impairs the function of insulin receptors in the brain, disrupts glucose and energy metabolism (13), and induces oxidative stress, neuroinflammation, cholinergic dysfunction, accumulation of tau protein and β-amyloid in the brain, extensive neuronal death, and cognitive deficits (14-16).

The anti-oxidant, anti-inflammatory, and anti-apoptotic properties of selenium (Se) have been documented in some tissues (17, 18); however, Se supplements typically exhibit low absorption rates and increased toxicity. Nanoparticle (NP) materials, such as selenium nanoparticles (SeNPs), offer an alternative form of nutritional supplementation with benefits, such as enhanced bioavailability, bio-activity, delivery, cellular uptake, and reduced toxicity (19, 20). Surface modification (coating) strategies for NPs can further improve their properties (21). Polyvinyl alcohol (PVA) is a water-soluble synthetic polymer utilized in coating various materials for industrial and medical purposes. Coating certain NPs with PVA has been shown to enhance their characteristics (22). Therefore, we investigated the effects of combined treatment with MSCs and SeNPs on rats with AD-like phenotypes. 

## Materials and Methods

Adult male Wistar rats (250±50 gr) were sourced from the breeding colony at Hamadan University of Medical Sciences. They were acclimatized to the laboratory condition at a consistent temperature of 23±2 °C and experienced a 12-hr light/12-hr dark cycle, with unrestricted access to water and food. The Ethics Committee of Hamadan University approved all treatments, animal care, and procedures (IR.UMSHA.REC.1398.629). Furthermore, all experiments were performed in compliance with the Guidelines for the Care and Use of Laboratory Animals (National Institutes of Health Publication No. 85–23, 1985).

***SeNPs-PVA preparation***

Selenium powder (0.25 M) was added to a sodium sulfite solution (0.50 M) in 100 ml of double distilled water and the mixture was stirred at 70 °C for 9 hr. This process yielded a transparent sodium selenosulfate solution, which served as a precursor for synthesizing SeNPs. The solution was then centrifuged (500 rpm, 20 min, 4 °C). An aqueous polyvinyl alcohol (PVA) stock solution, 1% by weight, was prepared by dissolving 1.0 g of PVA in 100 ml of water, with continuous stirring at 80 °C. PVA-stabilized SeNPs were synthesized by reacting sodium selenosulfate (concentration ranging from 1×10^−4^ to 1.0 × 10^−3^ mol l^-1^) with acetic acid (5 × 10^−3^ to 3 × 10^−2^ mol l^-1^) in an aqueous medium, with PVA acting as a stabilizer. In this step, SeNPs were distributed within the polymer matrix and stabilized, and the aggregation of nanoparticles was prevented. The formation of selenium NPs was observed within 30 sec. Without the PVA stabilizer, a precipitate of agglomerated selenium formed. 

***Cell culture***

Adipose tissue-derived mesenchymal cells (AMSCs) were isolated from male Wistar rats (aged 4–8 weeks) using the enzymatic digestion method. Initially, adipose tissue was excised from the abdomen of euthanized rats, washed with phosphate-buffered saline (PBS), finely minced, and transferred into a Falcon tube containing 1% collagenase. The adipose tissue was then incubated at 37 °C. After 1 hr, collagenase was added and following centrifugation, the supernatant was discarded. The resultant cells were cultured at a density of approximately 1×10^6. Finally, the cells were detached from the dish bottom through trypsinization.

***Characterization of SeNPs***

The morphology, structure, composition, and cytotoxicity of prepared SeNPs were determined using transmission electron microscopy (TEM), X-ray powder diffraction (XRD), Fourier-transform infrared spectroscopy (FTIR; Bruker Optics Ft Tensor, Germany), and MTT assay, respectively. 

Cell metabolic activity was measured using the MTT colorimetric assay (Thermo Fisher Scientific, USA). AMSCs were treated with different concentrations (0.01, 0.1, 1, and 10 μg/ml) of SeNPs for 24 hr. 

***Surgical procedures and STZ administration***

Animals were anesthetized with ketamine and xylazine (100 mg/kg and 20 mg/kg, Alfasan, Netherlands, respectively). A single unilateral cannula (10 mm, 23-gauge) was implanted into the ICV region (AP: −0.9 mm from the bregma; ML: +1.5 mm from the midline, and DV: −2.2 mm from the skull surface) following the rat atlas by Paxinos and Watson (23). The cannula was secured to the skull with two screws and dental cement. Following the procedure, animals were allowed to recover and then treated with an ICV injection of STZ on the first day, repeated on the third day (3 mg/kg, 10 μl per injection) (8).

***Experimental groups***

Forty-five rats were randomly divided into five groups (n =8-9 rats per group):

1. Control group: this group did not receive any treatment or surgery

2. Alz group: this group received the ICV injection of STZ 

3. Alz+ Stem cell group: Alz rats that underwent ICV transplantation of stem cells one month after STZ administration 

4. Alz+SeNP group: Alz rats that were treated orally with SeNP (0.4 mg/kg) for one month after STZ administration

5. Alz+stem cell+SeNP group: Alz rats that received both treatments (ICV transplantation of stem cells and oral administration of SeNP) 

***Novel object recognition test***

The novel object recognition (NOR) test was used to examine the recognition memory. Rodents naturally prefer exploring new environments and objects over familiar ones. The NOR test consists of three stages: habituation, training, and retention. During the habituation stage, animals acclimate to the environment. In the training phase, animals explore two identical objects for 10 min. Twenty-four hours later, the retention stage introduces both familiar and new objects for exploration, recorded over 10 min. Object exploration is considered when a rat sniffs, bites, or touches the objects. 

The discrimination index (DI) was assessed using the following formula (24):

$\mathrm{DI}=\frac{\mathrm{Novel object exploration time}-\mathrm{Total exploration time}}{\mathrm{Time spent exploring the novel objects}+\mathrm{Time spent exploring the familiar objects}}\times 100$

***Passive avoidance learning test***

Passive avoidance learning (PAL) was evaluated using a shuttle box apparatus, consisting of transparent plastic light (20 cm×20 cm×30 cm) and dark compartments (20 cm×20 cm×30 cm) separated by a guillotine door. The dark compartment featured a stainless grid floor. An electrical shock was administered to the floor of the dark compartment for 1.5 sec (25) by an electrified generator. 

The PAL test comprised three sessions, including habituation, learning, and reacquisition. Animals were first habituated to the apparatus, with the habituation process repeated 30 min later. During the learning stage, each rat was allowed to enter the dark compartment, and the latency to enter the dark compartment during acquisition (STLa) was recorded. Then, the guillotine door was closed and an electrical shock (0.4 mA, 50 Hz) was applied for 1.5 sec. After 2 min, the learning trial was repeated, administering another shock until the rat stayed in the bright compartment for 2 min. The number of shocks required to achieve this was noted (26).

The retention test occurred 24 hr after the learning trials. The rat was placed in the light compartment and, after 15 sec, the guillotine door was raised. The animal’s latency to enter the dark compartment (STLr) was recorded. Additionally, the duration spent in the dark compartment during the test was noted. 

***BDNF Measurement***

After completing the behavioral tests, the animals were anesthetized and euthanized, and their brains were promptly extracted from the skull and immersed in normal saline at 4 °C. The hippocampal tissues were then flash-frozen in liquid nitrogen. These tissues were combined and homogenized in phosphate saline buffer (pH 7.4), with a protease inhibitor cocktail added to prevent protein degradation. Subsequently, the samples were centrifuged at 4 °C and 10,000 rpm for 10 min. The supernatant was collected and stored at -80 °C for later analysis. The levels of BDNF protein were measured using the ELISA method, with specific kits and following the manufacturer’s instructions (ZellBio, Germany).

The peak absorption was recorded at 450 nm, and a standard curve was established. Protein concentration was determined using the Bradford method, with bovine serum albumin serving as the standard reference (6, 24).

***Measurement of oxidative stress markers***

*Total anti-oxidant capacity*

The ferric reducing anti-oxidant potential (FRAP) assay was used to measure the hippocampal total anti-oxidant capacity (TAC). Freshly prepared FRAP reagent and ferric chloride in distilled water were mixed with either the standard or test samples and incubated at room temperature. The absorbance of the samples was determined at 593 nm. The absorbance of each sample was compared to the standard curve, and the TAC was expressed in μmol/L (27). 

*Measurement of malondialdehyde*

The levels of malondialdehyde (MDA), the most important product of lipid peroxidation during oxidative stress, were determined using a thiobarbituric acid reagent (36). Briefly, 100 μl of serum was mixed with 200 μl of 8.1% SDS, 1.5 ml of 20% acetic acid, and 1.5 ml of 0.8% thiobarbituric acid. The mixture was heated in a boiling water bath for 60 min. After cooling, the mixture was centrifuged at 4000 rpm for 10 min. The supernatant was collected, and its absorbance was recorded at 532 nm. The MDA levels were calculated in μmol/l. 

*Measurement of the total thiol group*

Plasma total thiol groups (TTG) serve as markers of free radical damage and are sensitive to oxidative damage. To evaluate the total thiol level, we used 5,5′-dithiobis (2-nitrobenzoic acid) (Ellman reagent, DTNB). The thiol group reacted with the DTNB reagent, producing a yellow-colored complex with peak absorbance at 421 nm (27). 

***Statistical analysis***

Data analysis was done using the SPSS version 16.0 software package and one-way analysis of variance (ANOVA), followed by Tukey’s test. The data were expressed as mean + SEM. A *P*-value<0.05 was considered significant. 

## Results

***Scanning Electron Microscopy (SEM) analysis***

**Figure 1:**
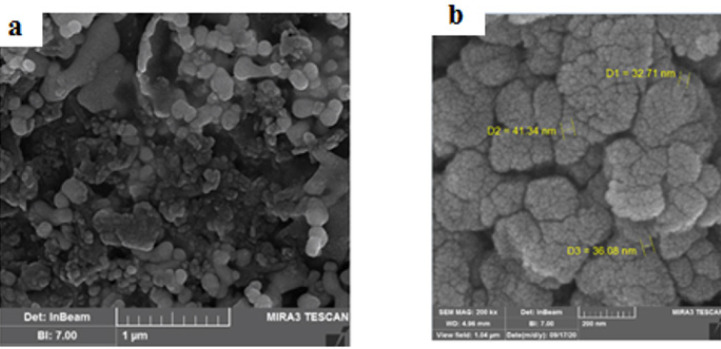
Image of selenium nanoparticles (SeNPs) by transmission electron microscopy. Scanning electron microscopy of selenium nanoparticles (a) and selenium polyvinyl alcohol nanocomposite (b)

SEM was applied to examine the surface morphology of SeNPs using a Hitachi S4160 apparatus. SEM analysis revealed a uniform surface structure of SeNPs, with the topographic structure appearing spherical, agglomerated, and uniformly distributed as shown in Figure 1. The images indicated that uncoated SeNPs were aggregated and compressed in the absence of PVA stabilizers. 

**SeNPs** ***characterization***

**Figure 2:**
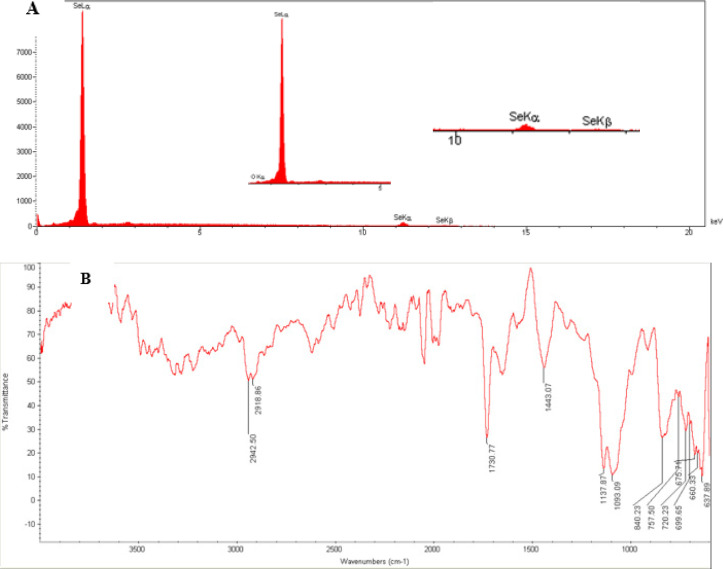
Characterization of selenium nanoparticles (SeNPs) by X-ray powder diffraction (A) and Fourier-transform infrared spectroscopy (B)

Figure 1 shows the morphology of SeNPs. The TEM technique revealed that SeNPs possess a spherical and monodisperse structure with diameters under 50 nm. X-ray Diffraction (XRD) analysis confirmed that the SeNP sample was nano-crystalline, with standard selenium powder successfully converted into SeNPs (Figure 2A). FTIR was used to analyze the SeNPs, covering a range from 400 to 4000 cm^-1^ (Figure 2B). 

**Figure 3:**
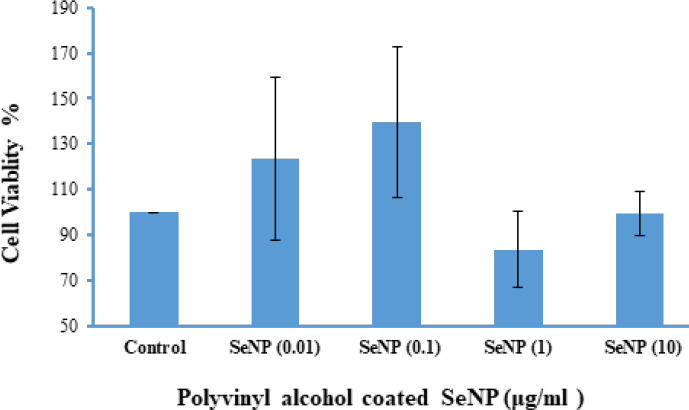
Comparison of different concentration of selenium nanoparticles (0.01, 0.1, 1, and 10 μg/mg SeNPs) on cell viability between experimental groups

Figure 3 illustrates the effect of different concentrations of SeNPs (0.01, 0.1, 1, and 10 μg/mg) on cell viability, indicating no significant differences between the groups. SeNPs did not exhibit any cytotoxic effects on cell viability, with different concentrations showing no toxic impact on AMSCs (*P*<0.001, Figure 3). 

***Behavioral assessments***

*NOR test*

**Figure 4:**
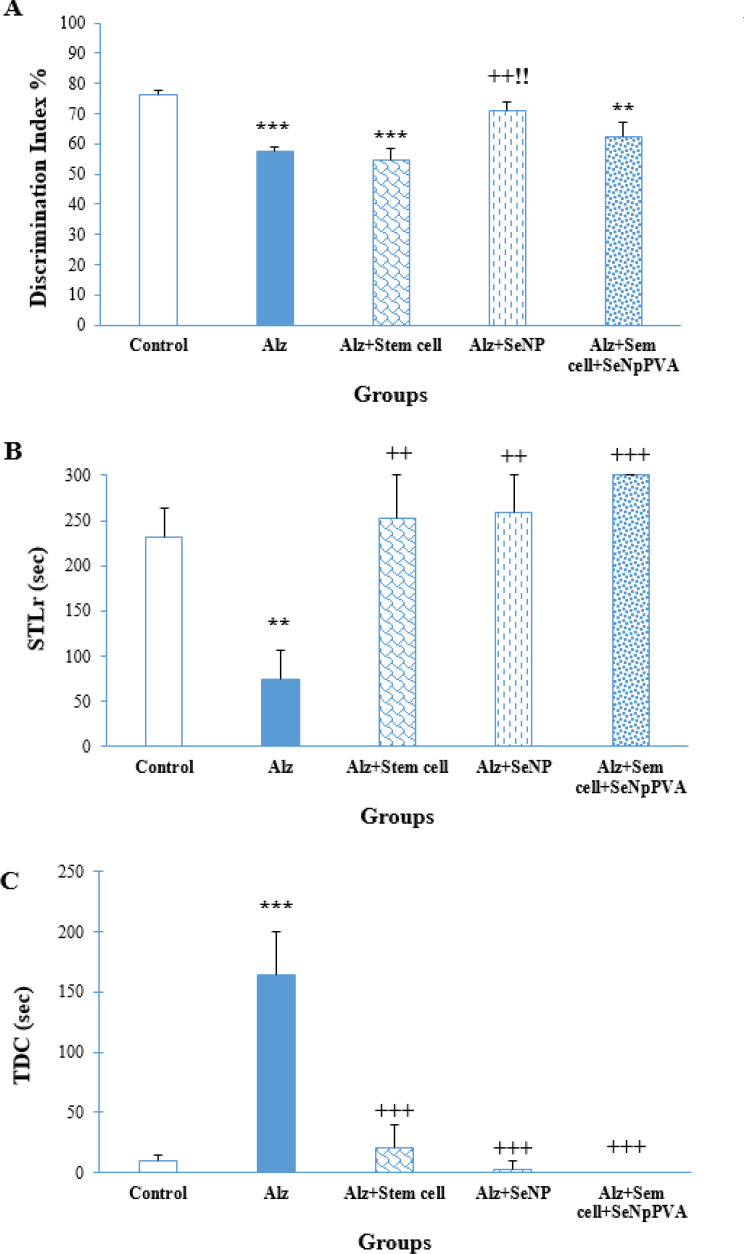
Effect of treatment with stem cells and selenium (Se) on the discrimination index in the object recognition test (A), step-through latency (STLr) (B), and time spent in the dark compartment (C) in the passive avoidance learning test.

Figure 4A illustrates the results of DI. Statistical analysis using one-way ANOVA showed significant differences between the groups (F (4, 42) = 13.806, *P*<0.001). According to Tukey’s *post hoc *test, it exhibited a reduced DI compared to both the control and Alz+SeNP groups (*P*<0.001 and *P*<0.01, respectively). Exhibited a reduced DI compared to both the control and Alz+SeNP groups (*P*<0.01). 

*PAL test*

Figure 4B indicates the results of STL in the reacquisition test. Significant differences were observed as indicated by one-way ANOVA (F (4, 42) = 7.262, *P*<0.001). Tukey’s *post hoc* test indicated that the Alz group had a reduced STLr compared to the control (*P*<0.01), Alz + stem cell (*P*<0.01), SeNP (*P*<0.01), and stem cell+ SeNP (*P*<0.001) groups. 

Figure 4C shows significant differences in TDC evidenced by one-way ANOVA (F (4, 42) = 11.242, *P*<0.001). Tukey’s *post hoc* test results showed that the Alz group had a shorter TDC compared to the control (*P*<0.001), Alz + stem cell + SeNP, and stem cell+ SeNP (*P*<0.001) groups. 

***Oxidative stress assessments***

**Figure 5:**
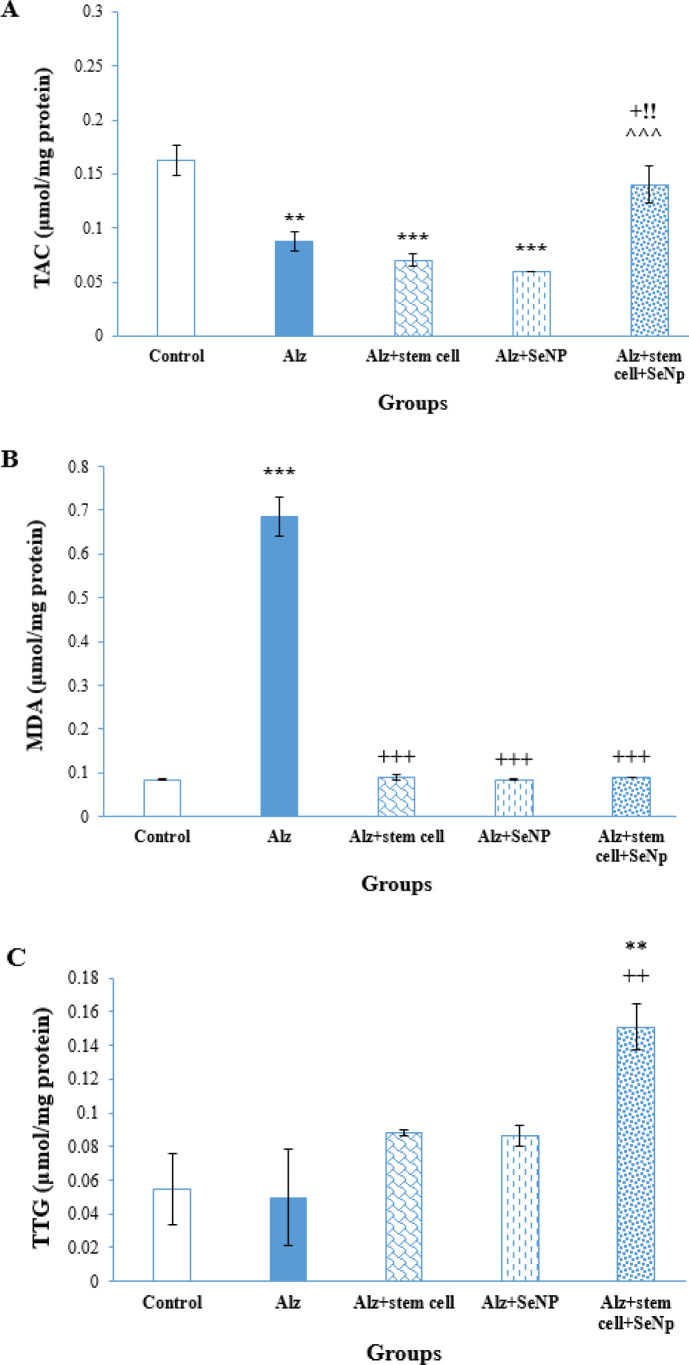
Comparison of oxidative stress indexes in the hippocampus of rats between experimental groups

TAC values and MDA and TTG levels were assessed to measure oxidative stress in the hippocampal cells of the rats. As depicted in Figure 5A, there was a significant difference in hippocampal TAC values between the groups [F (4, 19) =16.495, *P*<0.001]. The mean TAC values in the hippocampus of the Alz, ALZ+stem cell, and Alz+SeNP groups were significantly lower than that of the control group (*P*<0.001). The Alz+stem cell +SeNP group exhibited a significant increase in TAC values compared to the Alz and other treated groups (*P*<0.05 and *P*<0.001, respectively). Figure 5B illustrates the mean MDA levels, an indicator of lipid peroxidation in the hippocampus of the animals. According to one-way ANOVA results, there was a significant difference in MDA levels between the groups [F (6, 27) =175.315, *P*<0.001]. The hippocampal MDA levels significantly increased in the Alz group compared to the control, Alz+stem cell, Alz+SeNP, and Alz+stem cell+SeNP groups (*P*<0.001). There was no significant difference in MDA levels between the control and Alz groups. 

Figure 5C shows the mean TTG levels in the hippocampus of the groups. The mean TTG levels in the Alz+stem cell+SeNP rats were significantly higher compared to those of the control and other Alz-treated groups (*P*<0.01). There was no significant difference in TTG levels between the control, Alz+stem cell, and Alz+SeNP groups. 

***BDNF Levels***

**Figure 6:**
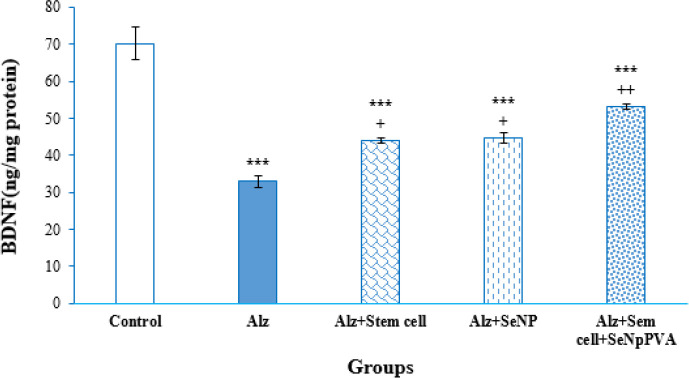
Measurements of brain-derived neurotrophic factor (BDNF) protein levels in the hippocampus of rats

Figure 6 shows the levels of BDNF protein in the hippocampus. According to the results of one-way ANOVA, a significant difference was observed in the BDNF levels between the groups [F (4, 19) = 35.9, *P*<0.001]. The Tukey *post hoc* test revealed a significant decrease in hippocampal BDNF levels in the Alz group compared to the control, Alz+stem cell, Alz+ SeNP, and Alz+stem cell+SeNP groups (*P*<0.001, *P*<0.01, *P*<0.01, and *P*<0.05, respectively). Also, the BDNF levels in the hippocampus of the control group were significantly higher compared to the Alz+stem cell, Alz+SeNP, and Alz+stem cell+SeNP groups (*P*<0.001).

*** *P*<0.001 and ** *P*<0.01 compared to the control group; +++ *P*<0.001 and ++(*P*<0.01) compared to the Alz group; and ++ *P*<0.01 compared to the Alz+ stem cell group. Each column represents mean + SEM.

Changes in total antioxidant capacity values (A), malondialdehyde (MDA) levels (B), and total thiol group (TTG) levels (C) in the rat's hippocampus

*** *P*<0.001 and ** *P*<0.01 compared to the control group; +++ *P*<0.001, ++ *P*<0.01, and + *P*<0.05 compared to the Alz group; !! *P*<0.01 compared to the Alz+ stem cell group; and ^^^ *P*<0.001 compared to the Alz+ stem cell+ SeNp group. Each column represents mean + SEM.

*** P<0.001 compared to the control group and ++ * P*<0.01 compared to the Alz group. Each column represents mean + SEM.

## Discussion

The present study evaluated the effects of co-treatment with PVA-coated SeNP and MSCs on STZ-induced AD-like phenotypes in rats, yielding the following findings: (a) ICV injection of STZ reduced TAC values and TTG and BDNF levels, and increased MDA levels in AD rats. The DI and reacquisition memory showed a decrease in the NOR and PAL tests in the ICV-STZ-treated group. (b) Treatment with MSCs in rats receiving STZ increased hippocampal BDNF levels, decreased MDA levels, and enhanced reacquisition memory in AD rats. (c) Administration of PVA-coated SeNP to AD rats resulted in increased hippocampal BDNF levels, decreased MDA levels, and improvement in cognition and reacquisition memory in the Alz group. (d) Co-treatment of AD rats with SeNP and MSCs increased hippocampal BDNF and TTG levels and TAC values, reduced MDA levels, and improved reacquisition memory. 

ICV injection of STZ has been widely employed to induce an AD model in experimental animal research (13). Currently, ICV-STZ administration has shown a decline in spatial memory and cognition, as demonstrated by changes in behavioral tests. Additionally, the results of our study indicated that ICV-STZ injection caused a decrease in hippocampal BDNF levels and oxidative damage in the hippocampus. Reduced BDNF signaling has been reported to lead to impaired memory and cognitive function (28). ICV injection of STZ likely induces oxidative stress by inhibiting ATP synthesis and acetyl-coenzyme A, consequently affecting acetylcholine synthesis (29). ICV STZ administration enters neurons via glucose transporters (GLUT2) (30) and induces pathophysiological occurrences such as aggregation of amyloid plaques in the cortex and hippocampus (24), and impaired synaptic plasticity (8).

At baseline, we evaluated the morphology and structure of the SeNPs. The sample and nanocrystalline synthesized by PVA-coated SeNP were confirmed by XRD pattern and FTIR spectrometer techniques. The cytotoxicity of SeNP on stem cells was assessed using the MTT technique. SeNPs exhibited low toxicity and inhibited cellular damage from free radicals (20). The results of the TEM technique showed that the NPs less than 50 nm were more favorable for cell culture (19). The CNS exhibits selective permeability through the blood-brain barrier, which restricts effective treatments for AD (3). NPs enhance drug delivery to neurons due to their small size (31). Se acts as an anti-oxidant, scavenging free radicals (32). SeNPs exhibit low toxicity, high biological availability, and the ability to cross the blood-brain barrier, thus facilitating drug treatment for brain diseases (33, 34). Furthermore, studies have shown that coated NPs display enhanced activity (35). Vinyl monomers serve as one of the coating agents to stabilize NPs (36). Polyvinyl alcohol (PVA) stabilizes the surface for Se (36, 37). The absorption of NPs and drug release from coated NPs are influenced by their density and electric charge, with coated NPs releasing drugs more slowly than uncoated ones (38). Therefore, PVA-coated SeNPs were utilized in the present study. 

Our findings are consistent with other research indicating that SeNPs mitigated oxidative stress in diabetic rats (39), reversed memory impairment, and exerted a neuroprotective effect in epileptic mice (40). Similar to nano-selenium, coated SeNPs mitigated toxic effects on rat brains through their anti-oxidant capacity (41) and also elevated BDNF levels in the hippocampus. 

NPs facilitate the transport of MSCs through the BBB. The impact of MSCs on memory has been documented in animal models of AD. The transplantation of MSCs led to memory improvements through an increase in TAC and BDNF levels (42). Our study verified that MSC transplantation in AD rats boosted BDNF production, diminished oxidative stress and Aβ expression, and enhanced learning and memory capabilities (43). 

The limitation of MSC therapy in treating AD lies in the death of transplanted stem cells in the new environment of the animal body (44). Utilizing NPs is an effective strategy to enhance the efficacy of stem cells. We observed that PVA-coated SeNPs enhanced the neuroprotective effects of transplanted MSCs in AD rats. The density and electric charge of NPs affect their absorption, and drug/agent release from coated NPs was slower than from uncoated ones (38). Combined treatment with PVA-coated SeNPs and MSC transplantation proved more effective in enhancing anti-oxidant capacity and increasing BDNF levels compared to AD groups that received only coated NPs or stem cell treatment. 

It has been reported that combination therapy has beneficial effects on neurodegenerative disorders (45). SeNPs have been shown to increase cell viability and differentiation of human embryonic stem cells into osteogenic cells *in vitro* and to facilitate neural differentiation in MSCs (46). Also, the combination of stem cells and NPs has been found to increase BDNF expression (47, 48) and improve neurological deficits in brain injuries (48) and memory impairment in a transgenic mice model of AD (47).

## Conclusion

In summary, our results showed that while each treatment alone improved memory, the combined administration of PVA-coated SeNPs and stem cells enhanced the therapeutic effects of each treatment. 
